# Fecal microbiota transplantation for intestinal rehabilitation after GI bleeding and perforation post–cardiac transplant: a case report

**DOI:** 10.3389/fmed.2026.1766362

**Published:** 2026-03-27

**Authors:** Yangyao Peng, Qian Hu, Caixia Gao

**Affiliations:** 1 Department of Cardiovascular Surgery, Zhongnan Hospital of Wuhan University, Wuhan, China; 2 Hubei Provincial Engineering Research Center of Minimally Invasive Cardiovascular Surgery, Wuhan, China; 3 Wuhan Clinical Research Center for Minimally Invasive Treatment of Structural Heart Disease, Wuhan, China; 4 State Key Laboratory of Metabolism and Regulation in Complex Organisms, Wuhan, China

**Keywords:** case report, fecal microbiota transplantation, gastrointestinal bleeding, heart transplantation, nutritional support

## Abstract

Gastrointestinal complications present a critical challenge following heart transplantation. These issues often stem from multifactorial mechanisms, including immunosuppressive therapy and physiological stress, which compromise mucosal defenses. We report a case of a 53-year-old heart transplant recipient who developed severe gastrointestinal bleeding and perforation due to stress ulcers. Following embolization therapy, the clinical course was further complicated by secondary intestinal cicatricial obstruction, necessitating effective intestinal rehabilitation. To address the resulting malabsorption and facilitate recovery while maintaining immunosuppressive stability, fecal microbiota transplantation (FMT) was employed to restore gut microbiota diversity. This intervention successfully promoted intestinal functional recovery. This case offers a practical reference for managing complex post-transplant gastrointestinal complications, highlighting the therapeutic potential of FMT.

## Introduction

Heart transplantation serves as the effective treatment for end-stage heart failure, significantly improving patients’ quality of life and prognosis (1, 2). However, postoperative management requires long-term immunosuppressive therapy and vasoactive support to maintain circulatory stability. These factors can compromise the gastrointestinal mucosal barrier, triggering stress ulcers or bleeding (3).

Despite routine prophylaxis with proton pump inhibitors, the occurrence of gastrointestinal bleeding or perforation poses a severe threat to transplanted heart function due to hemodynamic instability and increased infection risk. These complications are associated with decreased survival rates, prolonged hospital stays, and increased mortality (4). Notably, controlling such severe infections often necessitates the long-term use of broad-spectrum antibiotics.

While controlling infections, it inevitably disrupts the intestinal microecological balance, leading to severe gut microbiota dysbiosis (5). Gut microbiota dysbiosis has been confirmed to correlate with multiple adverse outcomes, including increased risk of opportunistic infections, immune regulation imbalance, and potential impacts on graft function and long-term survival (6).

Therefore, in the management of such patients, actively restoring the damaged microecology while minimizing antibiotic-induced disruption to gut microbiota has become a critical challenge in optimizing treatment strategies. This article reports a case of intestinal function recovery following fecal microbiota transplantation in a patient who developed gastrointestinal bleeding and perforation after heart transplantation. By analyzing this treatment process alongside literature review, we focus on discussing management strategies for such patients, aiming to provide insights into the application value and prospects of fecal microbiota transplantation for gut dysbiosis following heart transplantation.

## Case description

We report a case of a 53-year-old male admitted with end-stage heart failure on February 21, 2025. The patient underwent orthotopic heart transplantation on March 18, 2025. Postoperatively, the recovery was initially smooth; he was weaned off the ventilator by day 2 and tolerated a soft liquid diet. However, on March 25, 2025, the patient presented with recurrent melena and a positive fecal occult blood test (FOBT). Endoscopy confirmed gastrointestinal ulcer bleeding, which was managed via endoscopic hemostasis and interventional embolization therapy.

**Figure 1:**
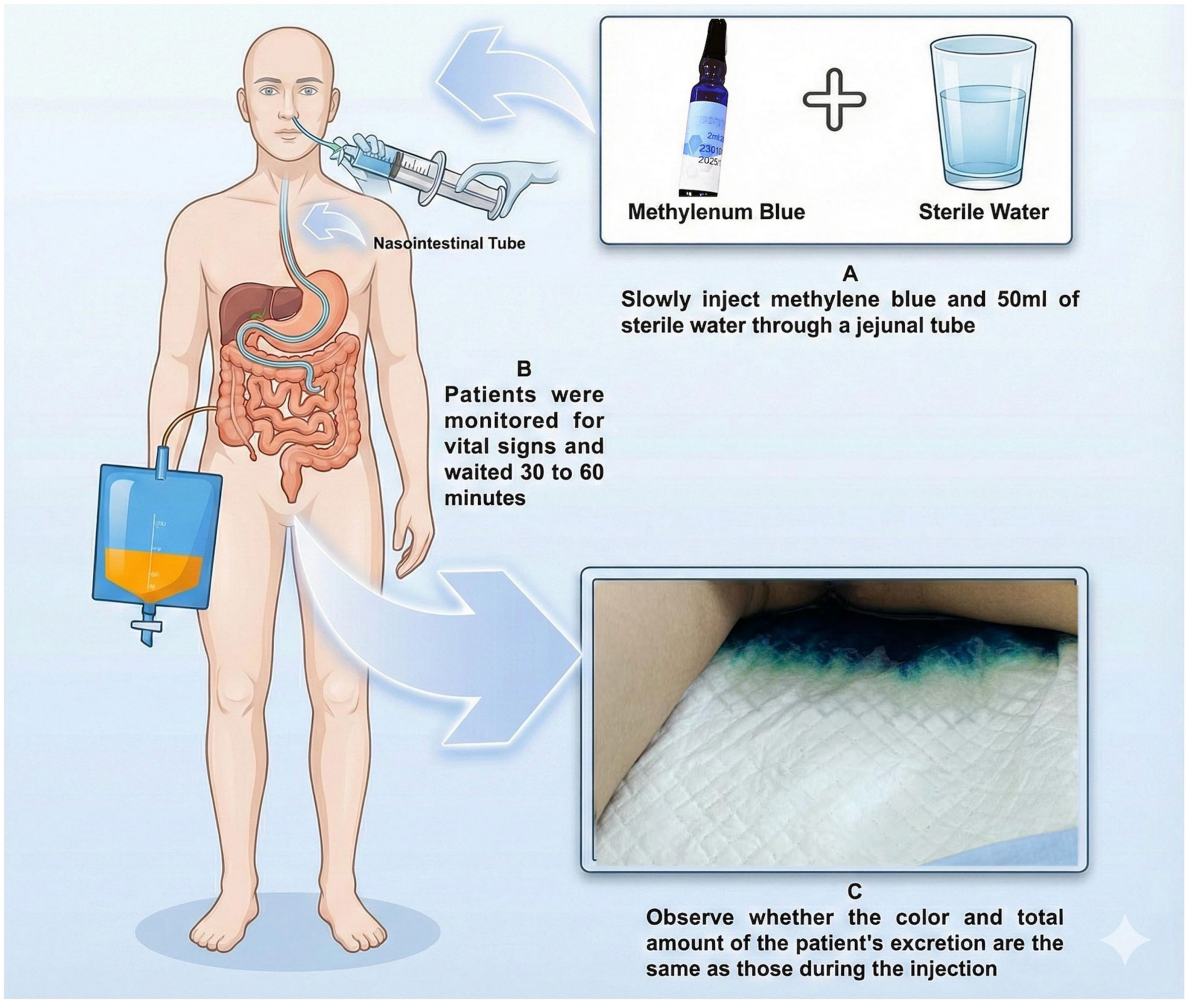
Injection methods and observations of methylene blue. **(A)** Methylene blue and 50 ml of sterile water are slowly injected through a jejunal tube. **(B)** Patients are monitored for vital signs during a 30 to 60-minute waiting period. **(C)** Observation of the patient’s excretion to determine if the color and total amount are the same as those during the injection.

Despite effective hemostasis, the clinical course was complicated by the development of an enterocutaneous fistula and secondary peritonitis. Following 44 days of comprehensive management—including anti-infection therapy, abdominal drainage, and nutritional support—the condition gradually improved, shifting the focus to intestinal rehabilitation. To verify fistula closure without the radiation exposure and infection risks associated with repeated CT scans, we employed a bedside methylene blue test. 50 mL of 2% methylene blue solution was instilled via nasogastric tube. The absence of dye in the abdominal drain, combined with its passage through the gastrointestinal tract (Figure 1), confirmed the restoration of intestinal continuity.

**Figure 2:**
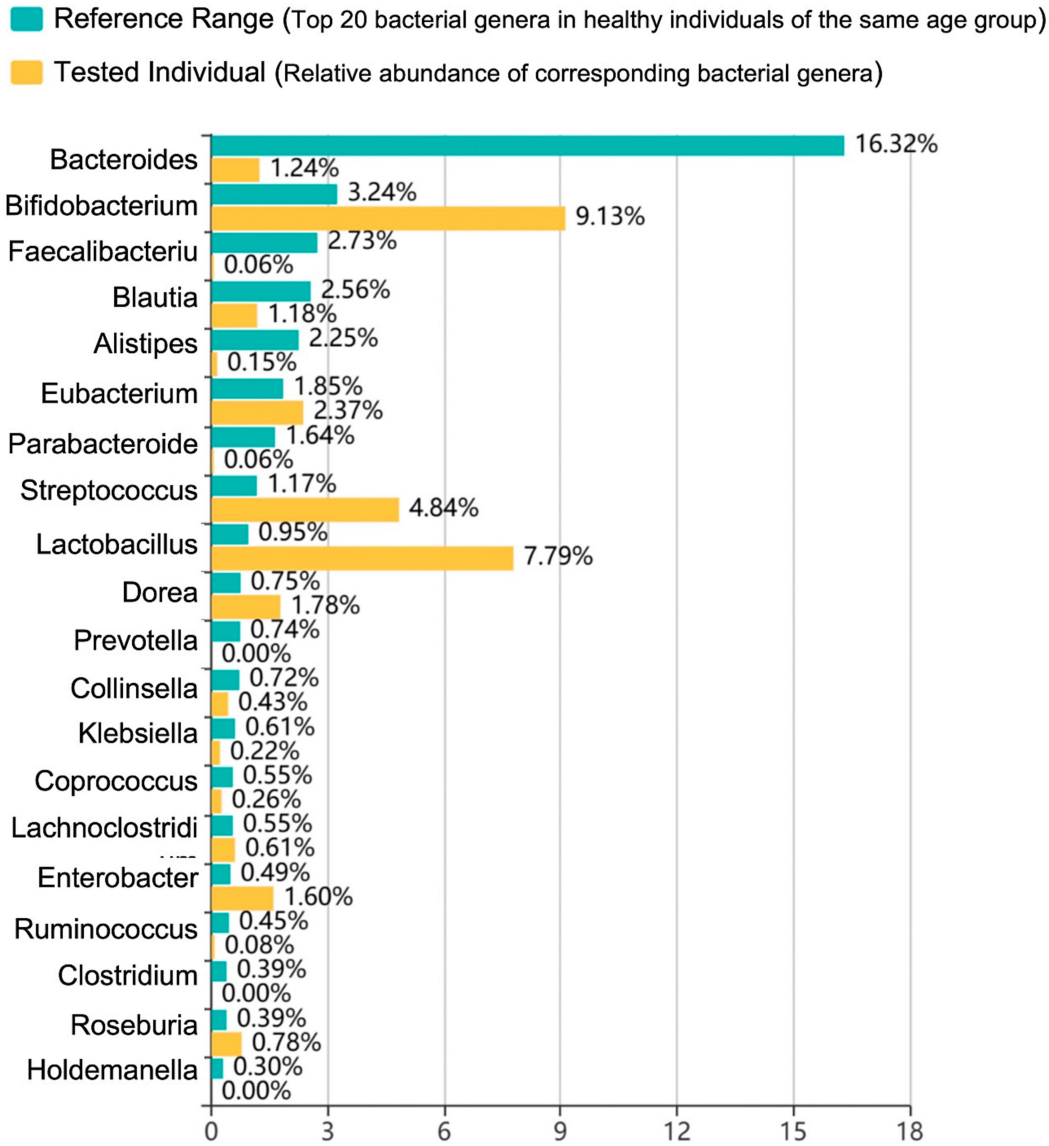
The abundance of gut microbiota.

Following 4 days of negative methylene blue testing, the procedure was concluded. However, prolonged fasting and antibiotic administration had disrupted the patient’s gut microbiota. Analysis of intestinal microbial diversity confirmed severe dysbiosis (Figure 2). To assess this, fecal samples were collected and total bacterial DNA was extracted. The composition of the gut microbiota was determined by sequencing the 16S rRNA gene. Taxonomic classification was subsequently performed to calculate the relative abundance of bacterial genera. The patient’s microbial profile was then evaluated against a reference range comprising the top 20 bacterial genera from healthy individuals of the same age group. This provided the indication for fecal microbiota transplantation (FMT) to reconstruct the intestinal microecology. Given the severity of the dysbiosis, we implemented an intensive therapeutic protocol: FMT was administered via nasogastric tube for 7 consecutive days. This standardized regimen aimed to maximize the colonization rate and accelerate the restoration of the mucosal barrier. FMT was administered according to standardized institutional protocol. Post-FMT, a stepwise nutritional support strategy was implemented, guided by the Bristol Stool Form Scale.

During the initial phase (Days 1 to 6), a restricted liquid diet (rice water, fish broth) and diabetes-specific enteral nutrition were introduced, while parenteral nutrition was maintained at 1000 mL/day. At this stage, the patient exhibited watery stools (Type 7) with high frequency (4–6 times/day). In the intermediate phase (Days 7 to 14), dietary volume was expanded to 800 mL/day with added vegetable juice, and enteral nutrition was escalated, while parenteral nutrition was concomitantly reduced. Consequently, stool consistency improved (Type 5) and frequency decreased. In the final phase (Days 15 to 29), parenteral nutrition was discontinued on Day 15, oral intake increased to 1,600 mL/day, and enteral nutrition was tapered off by Day 20.

**Figure 3:**
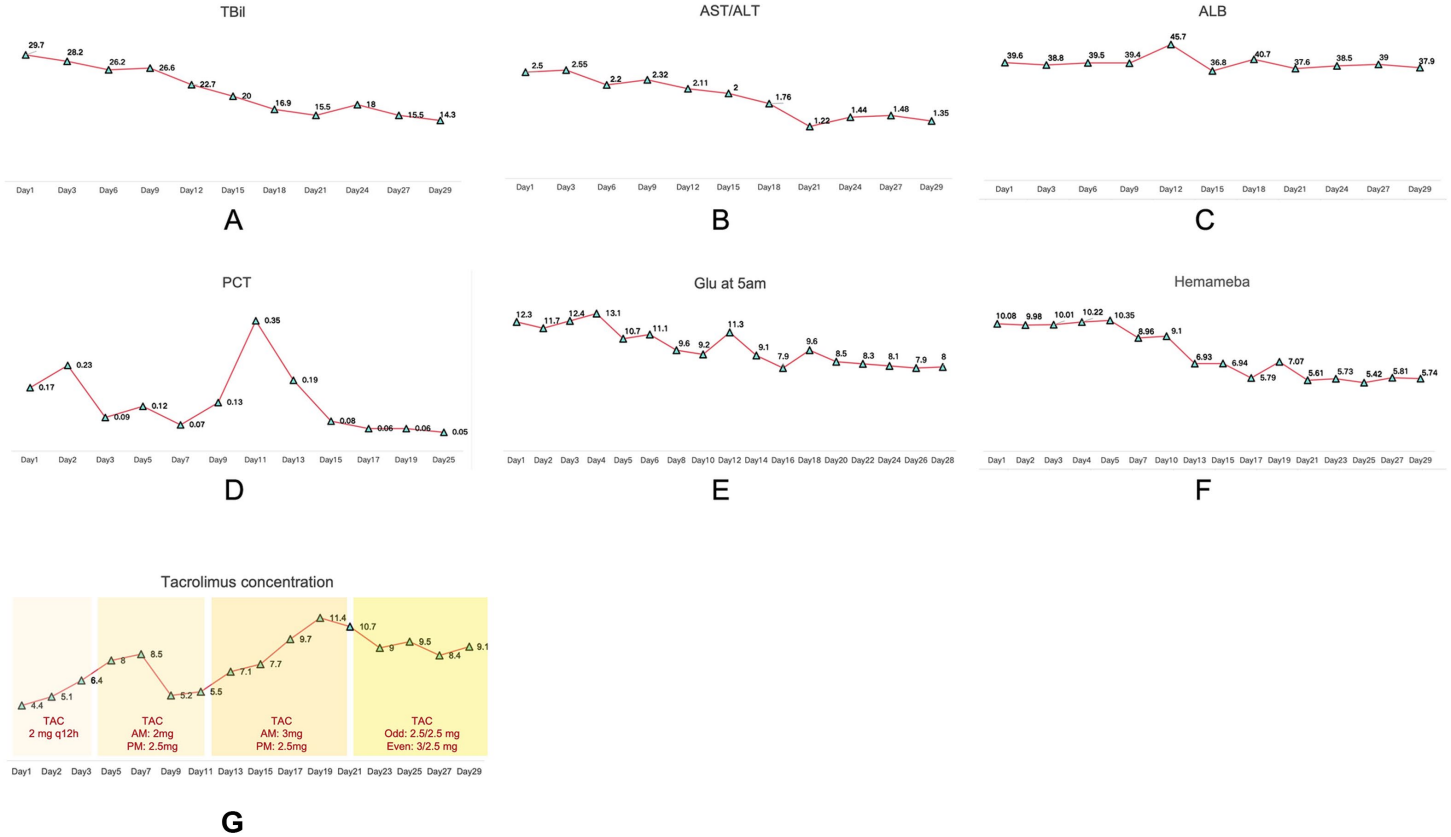
Test indicators after fecal microbiota transplantation. **(A)** TBil. **(B)** AST/ALT. **(C)** ALB. **(D)** PCT. **(E)** Glu at 5am. **(F)** Hemameba. **(G)** Tacrolimus concentration. TAC, tacrolimus; q12h, every 12 h. In the annotated text, “AM” and “PM” denote morning and evening doses, respectively. For the alternating schedule, “Odd” and “Even” indicate the specific doses administered on odd and even days.

By the treatment endpoint (Day 29), the patient achieved stable, well-formed soft stools (Type 4) with an ideal frequency of 1–2 times/day. This confirmed that the combined FMT and stepwise nutritional strategy effectively restored intestinal functional homeostasis (Table 1). Furthermore, inflammatory and homeostatic markers tracked from transplantation to discharge showed significant stabilization (Figure 3).

**Table 1:** Dietary and defecation conditions after intestinal flora transplantation.

Time	Diet management			Bowel movements	
	Daily diet	Enteral nutritional emulsion (1 Kcal = 1.1 mL)	Parenteral nutrition (1 Kcal = 1.4 mL)	Defecation traits	Frequency
Day 1	Rice soup, Fish soup (600 mL)	Fresubin diabetes (140 mL)	GAA-FE (1,000 mL)	Type 7	5
Day 2	Rice soup, Fish soup (600 mL)	Fresubin diabetes (150 mL)	GAA-FE (1,000 mL)	Type 7	6
Day 3	Rice soup, Fish soup (600 mL)	Fresubin diabetes (160 mL)	GAA-FE (1,000 mL)	Type 7	5
Day 4	Rice soup, Fish soup (600 mL)	Fresubin diabetes (170 mL)	GAA-FE (1,000 mL)	Type 7	5
Day 5	Rice soup, Fish soup (600 mL)	Fresubin diabetes (180 mL)	GAA-FE (1,000 mL)	Type 6	4
Day 6	Rice soup, Fish soup (600 mL)	Fresubin diabetes (190 mL)	GAA-FE (1,000 mL)	Type 5	2
Day 7	Rice soup, Fish soup, Vegetable juice (800 mL)	Fresubin diabetes (200 mL)	GAA-FE (1,000 mL)	Type 5	3
Day 8	Rice soup, Fish soup, Vegetable juice (800 mL)	Fresubin diabetes (250 mL)	GAA-FE (800 mL)	Type 5	3
Day 9	Rice soup, Fish soup, Vegetable juice (800 mL)	Fresubin diabetes (250 mL)	GAA-FE (800 mL)	Type 5	2
Day 10	Rice soup, Fish soup, Vegetable juice (800 mL)	Fresubin diabetes (250 mL)	GAA-FE (800 mL)	Type 5	2
Day 11	Rice soup, Fish soup, Vegetable juice (800 mL)	Fresubin diabetes (300 mL)	GAA-FE (600 mL)	Type 6	4
Day 12	Rice soup, Fish soup, Vegetable juice (800 mL)	Fresubin diabetes (300 mL)	GAA-FE (600 mL)	Type 5	2
Day 13	Rice soup, Fish soup, Vegetable juice (800 mL)	Fresubin diabetes (300 mL)	GAA-FE (600 mL)	Type 5	2
Day 14	Rice soup, Fish soup, Vegetable juice (800 mL)	Fresubin diabetes (300 mL)	GAA-FE (600 mL)	Type 4	2
Day 15	Rice soup, Fish soup, Vegetable juice (1,100 mL)	Fresubin diabetes (200 mL)	N/A	Type 4	1
Omit					
Day 20	Rice soup, Fish soup, Vegetable juice (1,200 mL)	N/A	N/A	Type 4	2
Omit					
Day 28	Rice soup, Fish soup, Vegetable juice (1,600 mL)	N/A	N/A	Type 4	2
Day 29	Rice soup, Fish soup, Vegetable juice (1,600 mL)	N/A	N/A	Type 4	1

## Discussion

To our knowledge, this represents the first case report describing the use of FMT for the treatment of persistent gut dysbiosis secondary to prolonged antibiotic therapy in a patient following cardiac transplantation. Given the ongoing dysbiotic state and its potential negative implications for immune regulation, infection risk, and graft function, we performed FMT for this patient. Post-treatment, the patient exhibited significant improvement in intestinal symptoms, restoration of gut microbiota diversity and beneficial bacterial abundance, and stabilization of the overall clinical condition, demonstrating the feasibility of utilizing FMT to correct intestinal dysfunction in the post-cardiac transplantation setting.

Fecal microbiota transplantation (FMT) has been established as the gold standard treatment for recurrent or refractory Clostridioides difficile infection (rCDI), with its core mechanism being the reversal of antibiotic-induced dysbiosis (7). In recent years, an increasing body of research has explored the application of FMT in non-CDI-associated antibiotic-related diarrhea and dysbiosis, particularly when conventional therapies have proven ineffective (8). FMT represents the primary ‘whole-gut microbiome replacement’ strategy and has been incorporated into clinical practice guidelines for the management of rCDI (9). Although this case did not represent typical rCDI, the underlying pathophysiology—characterized by severe, refractory dysbiosis resulting from prolonged broad-spectrum antibiotic use in an immunocompromised host—shares key pathophysiological features with rCDI.

In the management of this complex case, we prioritized FMT over probiotics or prebiotics due to the severity of the physiological disruption and the critical need for rapid intervention. While probiotics typically offer limited strain diversity and slower modulation, FMT facilitates the rapid reconstruction of the entire intestinal ecosystem, offering a more robust restoration of the mucosal barrier. This distinction is pivotal because the potential benefits of FMT extend beyond the immediate alleviation of gastrointestinal symptoms to potentially support long-term cardiac allograft stability. While FMT is not a direct treatment for rejection, it may confer significant indirect benefits via the “gut-heart axis.” Severe dysbiosis is known to trigger systemic inflammation, which can theoretically destabilize immune tolerance; by restoring healthy microbiota-host interactions, FMT helps modulate local and systemic immune status, thereby reducing the inflammatory burden on the graft. Moreover, a core challenge in these recipients lies in maintaining the stable pharmacokinetics of oral immunosuppressants (e.g., tacrolimus). Intestinal inflammation and dysbiosis can lead to malabsorption and erratic drug levels, inadvertently increasing the risk of rejection episodes. A previous foundational study (10) suggested that FMT combined with immunosuppressants may modulate allograft rejection. Our findings align with this, indicating that microbial reconstitution helps stabilize the metabolic kinetics of these agents. Critically, throughout the FMT treatment and subsequent follow-up period, the patient exhibited no clinical or subclinical signs of cardiac allograft rejection. This supports the hypothesis that restoring intestinal homeostasis is vital for maintaining a stable immunosuppressive state, although these deeper potential benefits merit further investigation.

The management of this case also highlights the unique challenges in monitoring strategies for cardiac transplant recipients with complex abdominal complications. Before successfully implementing FMT, it is crucial to ensure complete control of the intra-abdominal source of infection, namely the enteric fistula. However, monitoring such high-risk patients is inherently challenging. Although computed tomography (CT) and colonoscopy had definitively confirmed successful management of the initial gastrointestinal bleeding and closure of the primary fistula, concerns regarding potential recurrence or subtle leaks persisted. For transplant recipients under profound immunosuppression requiring protective isolation, repeated CT scanning carries significant drawbacks. On the one hand, cumulative radiation exposure poses a potential long-term risk for patients expected to achieve long-term survival who may face other health issues. On the other hand, frequent transportation of the patient out of the highly protected isolation unit to the radiology department substantially increases the risk of exposure to nosocomial pathogens (such as multidrug-resistant bacteria and fungi), an exposure that could be potentially catastrophic for an immunocompromised host.

Methylene blue, a safe, inexpensive, and readily available medical dye, has a long and extensive history of clinical use (11). Its novel application as a primary confirmatory tool—replacing repeated CT scans to exclude subtle leaks or confirm healing status in this extremely medically vulnerable population of cardiac transplant recipients—represents significant clinical innovation. The procedure was performed entirely at the bedside within the isolation unit, eliminating the need for patient transfer and thereby minimizing contact with the external environment and substantially reducing the risk of nosocomial pathogen exposure. The results—absence of dye in the effluent and its excretion—were immediate and visually evident. This provided rapid and reliable evidence to inform subsequent clinical decisions, such as permitting the initiation of enteral nutrition, considering FMT, and adjusting antibiotic therapy. Crucially, confirmation of fistula closure served as a paramount prerequisite for the safe implementation of FMT.

Furthermore, the immunological safety of FMT in profoundly immunocompromised cardiac transplant recipients warrants specific consideration. A primary theoretical concern is whether the sudden introduction of a complex foreign microbial community could inadvertently hyperactivate the suppressed immune system, potentially triggering allograft rejection or other immune-mediated complications. In our observation, the patient maintained stable cardiac graft function with no episodes of acute rejection or infectious complications following the FMT intervention. This suggests that in this specific instance, correcting severe dysbiosis may have facilitated immune homeostasis rather than causing destructive immune activation. However, it is crucial to emphasize that this report provides a clinical reference based on a single patient. Whether FMT poses other potential side effects, unexpected adverse reactions, or long-term immunological risks in this highly vulnerable population remains uncertain. The accumulation of more clinical experience and larger-scale cohort studies are highly warranted to fully establish its safety profile.

## Conclusion

The successful management of this case lies not only in the innovative application of FMT to address antibiotic-induced gut dysbiosis in a cardiac transplant recipient, and in confirming the paramount prerequisite of fistula closure, but also equally critically in the implementation of stringent safety protocols and the novel, minimally invasive methylene blue monitoring strategy to mitigate risks. Collectively, these elements represent an optimized, tailored, integrated management strategy for profoundly complex complications such as FMT in the post-cardiac transplant setting.

## Data Availability

The raw data supporting the conclusions of this article will be made available by the authors, without undue reservation.
